# Strong mechanical squeezing in an electromechanical system

**DOI:** 10.1038/s41598-018-21949-y

**Published:** 2018-02-23

**Authors:** Ling-Juan Feng, Gong-Wei Lin, Li Deng, Yue-Ping Niu, Shang-Qing Gong

**Affiliations:** Department of Physics, East China University of Science and Technology, Shanghai, 200237 China

## Abstract

The mechanical squeezing can be used to explore quantum behavior in macroscopic system and realize precision measurement. Here we present a potentially practical method for generating strong squeezing of the mechanical oscillator in an electromechanical system. Through the Coulomb interaction between a charged mechanical oscillator and two fixed charged bodies, we engineer a quadratic electromechanical Hamiltonian for the vibration mode of mechanical oscillator. We show that the strong position squeezing would be obtained on the currently available experimental technologies.

## Introduction

Nonclassical states^1,2^, as a very fundamental and practical application in quantum optics and quantum information processing, have attracted extensive attention. One of the most essential quantum states is the squeezed state^1,3,4^, in a harmonic oscillator, which can be defined as the reduction of uncertainty in one quadrature below the standard quantum limit at the expense of the corresponding enhanced uncertainty in the other, such that the Heisenberg uncertainty relation is not violated^5–8^. Since then, the schemes for producing and performing squeezed states have been intensively investigated via theoretical proposals and experimental implementations^9–34^.

Following the development of laser cooling of mechanical oscillators^35–38^, the preparations of mechanical squeezed states^10^ were widely used to study the applicability of quantum mechanics and the precision of quantum measurements^11,12^. In particular, the theoretical schemes for generation of the mechanical squeezing were proposed by amplitude-modulated driving field^16–18^, quantum measurement plus feedback^19,20^, two-tone driving^21^, injection of squeezed light^22^, or quadratic optomechanical coupling^23–30^. On the experimental side, the mechanical squeezing has been realized via reservoir engineering technique^32^, or parametric modulation^33^. The above-mentioned methods for preparation of the mechanical squeezing are based on optomechanical systems^10–12,15–30,32–34^, where via the radiation-pressure force, a laser-driven optical cavity is used to control a mechanical oscillator.

In this paper, we present an alternative scheme to effectively prepare strong mechanical squeezing in an electromechanical system, where via the Coulomb force, the coupling between a charged mechanical oscillator and two fixed charged bodies leads to the strong mechanical squeezing. Our proposed scheme in the electromechanical system has an important advance: the adjustment of the voltage of the bias gate could produce the large Coulomb force sufficient for the realization of the strong mechanical squeezing. The present results are applicable to generate the strong position squeezing of the mechanical oscillator, with presently available experimental capabilities.

## Results

**Figure 1:**
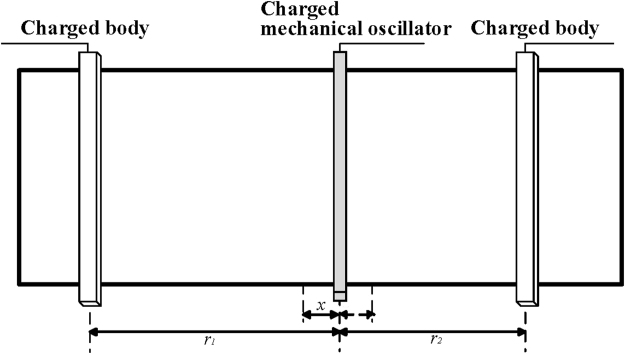
Schematic representation of the electromechanical system via the Coulomb force.

As shown schematically in Fig. 1, our model consists of a charged mechanical oscillator in the middle which is coupled to two fixed charged bodies on the left and right sides. The charged mechanical oscillator is subject to the Coulomb force due to the nearby charged bodies. The Hamiltonian describing the vibration of the charged mechanical oscillator is given by

1 $$H={H}_{0}+{H}_{{\rm{int}}},$$

with

2 $${H}_{0}=\frac{1}{2}m{\omega }_{m}^{2}{\hat{x}}^{2}+\frac{{\hat{p}}^{2}}{2m},$$

and^39–44^

3 $$\begin{array}{rcl}{H}_{{\rm{int}}} & = & {H}_{{\rm{int}}}^{(1)}+{H}_{{\rm{int}}}^{(2)}\\  & = & \frac{{C}_{0}{{\rm{U}}}_{0}^{2}}{2(1+\hat{x}/{r}_{1})}+\frac{{C}_{0}{{\rm{U}}}_{0}^{2}}{2(1-\hat{x}/{r}_{2})},\end{array}$$

here, *H*_0_ is the free Hamiltonian with $\hat{x}$ and $\hat{p}$ being the position and momentum operators for the vibration of the charged mechanical oscillator, with frequency *ω*_*m*_ and mass *m*, and ${H}_{{\rm{int}}}^{(l)}$ describes the Coulomb interaction between the charged mechanical oscillator and the *l*th (*l* = 1, 2) charged body. *C*_0_U_0_ is the positive charge on the charged mechanical oscillator, with *C*_0_ and U_0_ being the equilibrium capacitance and the voltage of the bias gate. *r*_*l*_ represents the equilibrium distance between the charged mechanical oscillator and the *l*th charged body.

In the case of $\hat{x}\ll {r}_{1},{r}_{2}$, the Coulomb interaction Hamiltonian ${H}_{{\rm{int}}}^{(1)}$ and ${H}_{{\rm{int}}}^{(2)}$ can be expanded to the second order of $\hat{x}/{r}_{l}$ as $\frac{{C}_{0}{{\rm{U}}}_{0}^{2}}{2}[1-\frac{\hat{x}}{{r}_{1}}+\frac{{\hat{x}}^{2}}{{r}_{1}^{2}}]$ and $\frac{{C}_{0}{{\rm{U}}}_{0}^{2}}{2}[1+\frac{\hat{x}}{{r}_{2}}+\frac{{\hat{x}}^{2}}{{r}_{2}^{2}}]$^45,46^. Considering *r*_1_ = *r*_2_ = *r*_0_ and omitting the constant term, we then obtain a simple form ${H}_{{\rm{int}}}=\frac{{C}_{0}{{\rm{U}}}_{0}^{2}}{{r}_{0}^{2}}{\hat{x}}^{2}$.

After defining dimensionless annihilation and creation operators for the vibration mode of mechanical oscillator using the position and momentum operators of the oscillator, $\hat{x}={[\hslash /(2m{\omega }_{m})]}^{\mathrm{1/2}}({\hat{b}}^{\dagger }+\hat{b})$ and $\hat{p}=i{[\hslash m{\omega }_{m}\mathrm{/2}]}^{\mathrm{1/2}}({\hat{b}}^{\dagger }-\hat{b})$, we rewrite the Hamiltonian *H* as

4 $$H^{\prime} =\hslash {\omega }_{m}{\hat{b}}^{\dagger }\hat{b}+\hslash g{({\hat{b}}^{\dagger }+\hat{b})}^{2},$$

where $g=\frac{{C}_{0}{{\rm{U}}}_{0}^{2}}{2m{\omega }_{m}{r}_{0}^{2}}$ is the effective mechanical coupling constant. In Eq. (4), we have neglected the zero-point energy from the first term. The second term is quadratic in position quadrature $\hat{x}$ of mechanical oscillator, which can produce quadrature squeezing through a unitary evolution on any initial state of mechanical mode. Note that the quadratic Hamiltonian *H*′ is similar to that of optomechanical systems for generating squeezing of the mechanical oscillator^23–30^. However in the quadratic optomechanical systems, the optomechanical coupling depends on average photons in the optical cavity. As the large photon numbers in the cavity tend to faster decay out of the cavity, the squeezing of the mechanical oscillator in refs^23–30^ is limited by the cavity decay.

Next, we consider the mechanical squeezing in the different temperatures of the environment. The state of the mechanical oscillator in thermal equilibrium with an environmental temperature *T* is described by means of the density matrix $\rho ={\sum }_{n}\,{p}_{n}|n\rangle \langle n|$, where ${p}_{n}=\mathrm{(1}-\exp [-\hslash {\omega }_{m}/{k}_{B}T])\,\exp (-n\hslash {\omega }_{m}/{k}_{B}T)$ represents the population in phonon number state |*n*〉 with *k*_*B*_ being the Boltzmann constant. In order to extract the squeezing properties of the mechanical mode, we need to calculate the mean square fluctuations 〈Δ*Q*(*t*)〉^2^ and 〈Δ*P*(*t*)〉^2^ ^20,30^ in the position and momentum of the mechanical oscillator. Let 〈Δ*Q*(*t*)〉^2^ = 〈*Q*(*t*)^2^〉 − 〈*Q*(*t*)〉^2^ and 〈Δ*P*(*t*)〉^2^ = 〈*P*(*t*)^2^〉 − 〈*P*(*t*)〉^2^, where $Q=1/\sqrt{2}({\hat{b}}^{\dagger }+\hat{b})$ and $P=i/\sqrt{2}({\hat{b}}^{\dagger }-\hat{b})$, satisfying the commutation relation [*Q*, *P*] = *i*. In Heisenberg picture, the operator *b* can evolve as the time-dependent operator^30^

5 $$b(t)=\exp [iH^{\prime} t/\hslash ]b\,\exp [-iH^{\prime} t/\hslash ]=rb\mathrm{(0)}+s{b}^{\dagger }\mathrm{(0)},$$

where exp[*iH*′*t*/*ħ*] is the time evolution operator, $r=\,\cos \,(qt)-\frac{if}{q}\,\sin \,(qt)$, $s=-\frac{2ig}{q}\,\sin \,(qt)$ with *f* = 2*g* + *ω*_*m*_ and $q=\sqrt{{f}^{2}-4{g}^{2}}$. Thus we can obtain ${\langle {\rm{\Delta }}Q(t)\rangle }^{2}=\frac{V}{2}[1-\frac{4g}{4g+{\omega }_{m}}\,{\sin }^{2}\,(qt)]$ and ${\langle {\rm{\Delta }}P(t)\rangle }^{2}=\frac{V}{2}\,[1+\frac{4g}{{\omega }_{m}}\,{\sin }^{2}\,(qt)]$, where $V=2{\bar{n}}_{th}+1$ with ${\bar{n}}_{th}={[\exp (\hslash {\omega }_{m}/{k}_{B}T)-1]}^{-1}$ being the mean number of thermal excitation phonons. At *qt* = *π*/2, 〈Δ*Q*(*t*)〉^2^ and 〈Δ*P*(*t*)〉^2^ become minimum and maximum as $\frac{V}{2}\frac{{\omega }_{m}}{4g+{\omega }_{m}}$ and $\frac{V}{2}\frac{4g+{\omega }_{m}}{{\omega }_{m}}$, respectively. Note that the product of the mean square fluctuations (〈Δ*Q*(*t*)〉^2^)_min_ and (〈Δ*P*(*t*)〉^2^)_max_ is *V* ^2^/4. In the particular case of the vacuum state, the position and momentum variances ${\langle {\rm{\Delta }}Q\rangle }_{vac}^{2}$ and ${\langle {\rm{\Delta }}P\rangle }_{vac}^{2}$ are 1/2. The degree of the squeezing S in units of decibel (dB) can be calculated by $-10\,{\mathrm{log}}_{10}\frac{{({\langle {\rm{\Delta }}Q(t)\rangle }^{2})}_{{\rm{\min }}}}{{\langle {\rm{\Delta }}Q\rangle }_{vac}^{2}}=-10\,{\mathrm{log}}_{10}\frac{V}{1+4g/{\omega }_{m}}$^47^. Clearly, the squeezing is primarily controlled through the coupling constant *g*, the mechanical frequency *ω*_*m*_, and the temperature *T*.

## Discussion

**Figure 2:**
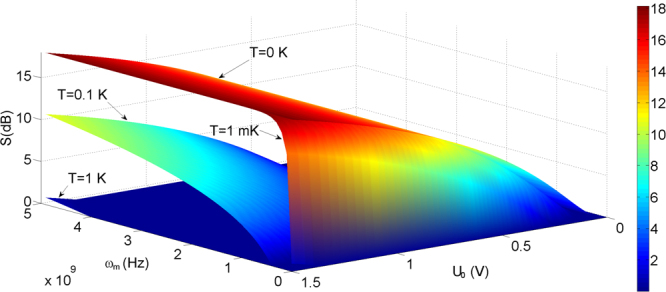
The degree of the squeezing S versus the frequency and the voltage for different temperatures of the environment *T* = 0 K, 1 mK, 0.1 K, 1 K when *C*_0_ = 5 nF, *r*_0_ = 4 *μ*m, and the elastic coefficient $k=m{\omega }_{m}^{2}=22$ N/m.

The degree of the squeezing S as functions of the mechanical frequency (*ω*_*m*_ = 5 MHz–5 GHz) and the voltage of the bias gate (U_0_) for different temperatures of the environment *T* = 0 K, 1 mK, 0.1 K, 1 K when *C*_0_ = 5 nF, *r*_0_ = 4 *μ*m, and the elastic coefficient $k=m{\omega }_{m}^{2}=22$ N/m is shown in Fig. 2. It is observed that at low temperature, the adjustment of the voltage of the bias gate could yield the strong squeezing in the high frequency. For example, we choose the realistic parameters corresponding to the experiment *ω*_*m*_ = 5.6 MHz and *m* = 0.7 ng^44^. Using *C*_0_ = 5 nF, U_0_ = 10 V, and *r*_0_ = 4 *μ*m, we obtain *S* ≈ 14.8 dB at the equilibrium temperature *T* = 1 mK and the evolution time *t* = *π*/(2*q*) ≈ 5.2 ns. According to ref.^13^, there exists a critical time ${t}_{diss}=\mathrm{1/}(\gamma {\bar{n}}_{tot})$, here *γ* is the mechanical damping rate and ${\bar{n}}_{tot}\approx {\sinh }^{2}\,\mathrm{(2}\xi )+{\bar{n}}_{th}$ is the total phonon number with *ξ* being the squeeze parameter. Choosing the damping rate *γ* = 204 Hz^44^, we get *t*_*diss*_ ≈ 0.16 ms, which satisfies the condition $t\ll {t}_{diss}$, and thus the decoherence of the mechanical oscillator could be negligible.

## Conclusion

In summary, we have proposed an effective method to generate the mechanical squeezing in the electromechanical system. This is realized through the Coulomb interaction acting on the charged mechanical oscillator and two charged bodies, implementing the strong squeezing of the mechanical oscillator. It is found that at low temperature, the squeezing can be enhanced by moderately increasing the voltage of the bias gate. Our proposed scheme would contribute to the experimental study of fundamental aspects in the macroscopic quantum effects and the precision of quantum measurements with mechanical oscillators.
